# Clustering of physical activity, sedentary behavior, and diet associated with social isolation among brazilian adolescents

**DOI:** 10.1186/s12889-023-15444-x

**Published:** 2023-03-25

**Authors:** Thiago Sousa Matias, Julianne Fic Alves, Gislaine Terezinha Amaral Nienov, MarcusVinicius Veber Lopes, Diego Itibere Cunha Vasconcellos

**Affiliations:** 1 Department of Physical Education, Federal University of Santa Catarina, Florianópolis, 88040-900 Brazil; 2 Institute for Positive Psychology and Education, Australian Catholic University, Sydney, Australia

**Keywords:** Healthy Lifestyle, Loneliness, Mental Health, Health Surveys, Motor Activity

## Abstract

**Backgound:**

Although obesogenic behaviors have been found to be related to social isolation, evidence-based person-centered approaches are lacking. This study investigated the association between clusters of obesogenic behavior – derived from a data-driven process – and social isolation among Brazilian adolescents.

**Methods:**

Data from the National Adolescent School-based Health Survey (PeNSE) 2015 were analyzed. A total of 100,794 9^th^-grade students (51.3% females; 14.3 ± 0.1 years old) enrolled in 3,040 public and private high schools participated in the study. Social isolation was assessed by two outcomes (i.e., perceived loneliness and lack of close friends). A two-step cluster analysis was conducted to identify patterns of obesogenic behaviors with the input of leisure-time physical activity (PA), sitting time as a proxy of sedentary behavior (SB), and the weekly consumption of healthy and unhealthy food. Crude and adjusted binary logistic regression models were applied to evaluate the associations between the clusters of obesogenic behaviors and social isolation variables in adolescents.

**Results:**

Three clusters were identified. Adolescents in the “Health-promoting SB and diet” (32.6%; OR = 0.69; 95% CI = 0.62–0.76) and “Health-promoting PA and diet” (44.9%; OR = 0.73; 95% CI = 0.67–0.79) clusters had lower odds of loneliness compared to those in the “Health-risk” cluster (22.5%). Those belonging to the “Health-promoting PA and diet” cluster were more likely to report having close friends (OR = 1.19; 95% CI = 1.00–1.41) than those in the “Health-risk” cluster.

**Conclusion:**

Adolescents in clusters where positive behaviors outweighed negative ones were less likely to perceive themselves as lonely and without close connections.

**Supplementary Information:**

The online version contains supplementary material available at 10.1186/s12889-023-15444-x.

## Introduction

Regular practice of physical activity (PA) and reduced time in sedentary behaviors (SB) have been positively associated with mental health in adolescence [1, 2]. This benefit includes favoring socialization and avoiding social isolation [3, 4]. Thus, recent evidence has shown that both high PA and low SB are associated with lower loneliness [5]. Active adolescents can be more socially integrated [4], while having friends help them to overcome barriers associated with a less active lifestyle [6].

It is important to consider that PA and SB do not occur in isolation in adolescents’ lives. These behaviors carry synergies with other behaviors such as diet, for example, which can (synergically speaking) influence socialization [6]. This influence might depend on the extension of adolescents' lifestyle have more favorable than unfavorable behaviors coexisting [7, 8]. These behaviors can be more or less obesogenic depending on the different profiles observed [3].

The problem that arises is that: (a) being in an obesogenic cluster can make socialization difficult, increasing social isolation [9, 10]; and (b) clusters that combine positive and negative behaviors can still favor better socialization and psychological disposition in adolescents when compared to clusters that mostly combine risk behaviors [2, 11]. The pioneering study by Iannotti and Wang (2013) [11], which investigated clusters of obesogenic behaviors and their impact on physical and mental health in adolescents in the United States, found that the group with the highest sedentary behavior and the highest proportion of non-healthy diet, but who still moderately met physical activity criteria or consumed fruits and vegetables daily, had lower levels of body dissatisfaction. Similarly, Matias and colleagues reported that adolescents in healthier clusters were more likely to be satisfied with their body image [2]. More recently, a large survey of Brazilian adolescents found that obesogenic clusters with more positive behaviors than negative ones were associated with prosocial attitudes and reduced adolescent bullying [12].

Despite some evidence from cross-sectional studies, few investigations have explored the association between clusters of obesogenic behaviors and psychosocial factors, such as social isolation. It is important to note that adolescents do not possess isolated virtues in their lifestyle, and there is an interplay between essentially positive or negative behaviors that can have an impact on mental health in numerous ways. Therefore, countries with low- to middle-income, like Brazil, have undergone demographic and epidemiologic transitions characterized by economic and sociocultural disparities, significant increases in inequality [13], and a corresponding rise in diseases associated with changes in lifestyle. Thus, the aim of the present study was to investigate the association between clusters of obesogenic behaviors and social isolation in a population-based study of Brazilian adolescents.

## Methods

### Study design and participants

A cross-sectional study using data from the National Adolescent School-based Health Survey (PeNSE – sample 1) was conducted in 2015 by the Brazilian Institute of Geographic and Statistics and the Ministry of Health of Brazil. PeNSE relates to World Health Organization recommendations for health surveys among students. The study investigates adolescents’ health and lifestyle behaviors among a nationally-representative sample of students enrolled in the 9^th^ grade of elementary school from public and private schools. PeNSE sampling process was planned to represent all geographical areas of Brazil. A total of 102,301 students among 3,040 schools were initially assessed; 229 students declined to participate or did not report their age or sex. The sampling strategy included geographical stratification and the multi-stage selection that can be seen elsewhere (Oliveira et al., 2017). Ethical approval was obtained, and the participation of all subjects was approved by the National Committee of Ethics in Research *(Comissão Nacional de Ética em Pesquisa [Conep])* number 1.006.467/2015. Methods were performed in accordance with the relevant guidelines and regulations.

The present survey is in its third edition. The questionnaire used for data collection is based on the Global School-Based Student Health Survey and Youth Risk Behavior Surveillance System and has been tested and adjusted [14].

### Clusters formation

To cluster formation, leisure-time physical activity (PA), sedentary behavior (SB), and diet were analyzed. Students’ PA was assessed using the question: In the past 7 days, without considering physical education class, how many days did you practice some physical activity like sports, dance, gym exercises, combat sports or other activity? The answers ranged from none to seven days in a week. SB was reasonable during the sitting time using the question: In a regular day, how much time do you spend watching television, playing video games, talking with friends or other sitting activities? The response options ranged from one to nine hour a day. Diet was assessed as continuous scores related to the weekly consumption of healthy (green salads or vegetables and fruits) and unhealthy food (deep-fried empanadas, candies, soda, fast foods, and ultra-processed food). Details on measurement, clusters formation procedure, and description have been provided elsewhere [8].

Briefly, a two-step cluster analysis was employed using the log-likelihood as the distance measure (to account for the congruency between clusters). Leisure-time PA, SB, and both healthy and unhealthy diet scores were independently included as continuous variables in the model. The low Schwarz’s Bayesian Criterion (BIC), the high ratio of distance measures, and the high ratio of BIC changes were used to determine the number of clusters. The theoretical assumption regarding the acceptability of the profiles was taken into account. The analysis was replicated among younger and older adolescents to check clusters’ acceptability [8]. Adolescents that have incomplete or missing data for PA, SB, or diet were not analyzed (*n* = 1,507, < 1.5% of the total sample).

100,794 adolescent students were distributed into three different profiles: The “Health-promoting SB and diet,” comprising 32.6% of the sample; the “Health-promoting PA and diet” (44.9% of the sample), the “Health-risk” cluster containing 22.5% of the sample. The two health-promoting clusters are those where positive behaviors prevail over the negative ones, and the health-risk cluster combines a negative profile for all variables (see Table 1). The process of cluster labeling was arbitrary and aimed to give a more intuitive interpretation of the results. The assumptions of "healthy" or "risk" were based on the z-score of each variable imputed in the model. The cluster names were an overall idea of a possibly “more healthy” or “more unhealthy” pattern. Therefore, the labeling does not represent standardized cut-off points. A detailed description of the clusters can be seen elsewhere [8].

**Table 1:** Physical activity (PA), sedentary behavior (SB), unhealthy diet, and healthy diet in each of the three clusters. National School-Based Health Survey among ninth-grade students—PeNSE, Brazil 2015 (sample 1)

	Health-promoting SB and diet Mean ± SD	Health-promoting PA and diet Mean ± SD	Health-risk Mean ± SD
Physical activity (day/week)	0.68 ± 0.92	4.56 ± 2.05	0.86 ± 1.24
Sedentary behavior (hour/day)	2.59 ± 1.55	3.85 ± 2.31	7.78 ± 1.39
Unhealthy diet (day/week)	1.68 ± 0.97	2.87 ± 1.49	3.24 ± 1.45
Healthy diet(day/week)	2.68 ± 1.97	4.10 ± 2.07	2.58 ± 1.97

### Social isolation

Participants were asked about two aspects: (a) “In the past 12 months, how often have you felt alone”. A five-point Likert scale ranging from never to always was the response option. Those who responded always or most of the time were considered lonely; (b) “How many close friends do you have?”. The option of response was zero/one/two/three or more. Those who responded “zero” were considered to having no close friends.

### Covariates

The covariates were: sex, skin color, age, live with mother, live with father, mother's schooling, residents of the house, cigarette smoking, alcohol consumption, drugs consumption, physically aggression by an adult at home, involved in a fight with a firearm, involved in a fight with a melee weapon, suffered physical aggression, got involved in a fight, body satisfaction, health perception, type of school.

### Statistical analysis

The participants’ characteristics were described using absolute and relative frequency with 95% confidence intervals (95%CI) for nominal variables and means with standard deviations (SD) for numerical variables. Crude and adjusted binary logistic regression models were applied to evaluate the associations between exposure (the clusters) and social isolation variables (perceived loneliness and having close friends). For each model, a set of covariates was selected based on empirical and theoretical evidence [12] (see tables notes); The Rao Scott chi-square test were performed to evaluate the association of each covariate and both outcomes of social isolation. Significant predictors at *p*-value < 0.2 were retained and included in the adjusted models. Collinearity was examined using the Variance Inflation Factor (VIF). No evidence of multicollinearity was found as the VIF values for all covariates were small (< 5). The “Health-risk” cluster was the reference category in the regression models. The results were expressed in odds ratios (OR) and the respective (95% CI). All inferential procedures included the survey design and weighting. Data were analyzed using STATA 15 software (Stata Inc., College Station, TX, USA), except for cluster procedures. The significance level was defined as *p* < 0.05.

## Results

Table 2 shows the characteristics of the participants. A total of 100,794 students with a mean age of 14.28 years old (SD = 0.013) were observed. Regarding social isolation, 16.39% of the adolescents experienced loneliness (95% CI = 15.93–16.85) and 4.29% reported not having any close friends (95% CI = 4.05–4.54). Other individual aspects such as behavioral characteristics, victimization, and health outcomes, are presented in the supplemental material.

**Table 2:** Characteristics of the sample. PENSE, Brazil 2015 (*n* = 100,794)

Variables	n	%	95% CI
***School-level covariates***			
Type of school			
Municipal	288	0.1	0.04—0.26
State	49,462	48.35	45.05—51.66
Federal	31,404	37.09	34.15—40.13
Private	20,918	14.46	12.53—16.63
Full-time school			
Yes	22,854	22.07	21.19—22.98
No	78,715	77.93	77.02—78.81
Boarding school			
Yes	3,846	4.085	3.748—4.451
No	97,942	95.92	95.55—96.25
***Household-level covariates***			
Computer at home			
Yes	69,822	69.56	68.51—70.6
No	32,144	30.44	29.4—31.49
Internet at home			
Yes	78,395	77.55	76.69—78.38
No	23,572	22.45	21.62—23.31
Household residents (mean)	–	4.49	4.47—4,52
***Sociodemographic factors***			
Sex			
Male	49,290	48.72	48.09—49.34
Female	52,782	51.28	50.66—51.91
Skin Color			
White	33,775	36.15	35.12—37.18
Black	12,849	13.39	12.88—13.92
Yellow	4,580	4.11	3.87—4.36
*Pardo*	46,935	43.05	42.17—43.94
Indigenous	3,825	3.29	3.07—3.53
Age, mean	–	14.29	14.26—14.31
Mother's schooling			
Did not studied	5,531	5.38	5.089—5.695
Incomplete elementary school	18,217	19.38	18.8—19.97
Elementary School	6,024	6.465	6.167—6.775
Incomplete high school	6,275	6.05	5.78—6.33
High school	17,903	18.01	17.47—18.57
Incomplete college	5,456	4.54	4.27—4.83
College	17,232	13.26	12.34—14.23
Do not know	25,183	26.9	26.25—27.56
Live with mother			
Yes	90,458	89.94	89.57—90.3
No	11,543	10.06	9.7—10.43
Live with father			
Yes	63,600	63.71	63.01—64.41
No	38,341	36.29	35.59—36.99
Has a cell phone			
Yes	88,978	87.38	86.87—87.87
No	13,012	12.62	12.13—13.13

*n*  Absolute frequency, *%*  Prevalence; *95% CI* 95% Confidence interval 95%

Table 3 shows the association between clusters and social isolation variables among adolescents. In the adjusted analysis, adolescents in the Health-promoting SB and diet (OR = 0.69; 95% CI = 0.62–0.76) and in the Health-promoting PA and diet (OR = 0.73; 95% CI = 0.67–0.79) clusters showed reduced odds of loneliness compared to those in the Health-risk cluster. Those belonging to the Health-promoting PA and diet cluster were more likely to have close friends (OR = 1.19; 95% CI = 1.01–1.41) compared to those in the health-risk cluster.

**Table 3:** Associations between the clusters and the perception of loneliness and friendships among the adolescents. PeNSE, Brazil 2015

	Perceived loneliness (*n* = 98,485)		Friendship (*n* = 97,898)	
	Crude	Adjusted^a^	Crude	Adjusted^b^
	OR (95% CI)	OR (95% CI)	OR (95% CI)	OR (95% CI)
**Clusters**				
Health-risk	Ref	Ref	Ref	Ref
Health-promoting SB and diet	0.50 (0.46—0.55)	**0.69 (0.62—0.76)**	0.89 (0.76—1.03)	0.86 (0.74—1.02)
Health-promoting PA and diet	0.50 (0.46—0.55)	**0.73 (0.67—0.79)**	1.23 (1.04—1.45)	**1.19 (1.002—1.41)**

*OR* Odds Ratio, 95% *CI* 95% Confidence Interval; Estimates were weighted according to the sampling design; ^a^Adjusted for sex, skin color, age, live with mother, live with father, mother's schooling, residents of the house, cigarette smoking, alcohol consumption, drugs, physically aggression by an adult at home, involved in a fight with a firearm, involved in a fight with a melee weapon, suffered physical aggression, got involved in a fight, body satisfaction, health perception, type of school. ^b^Adjusted for sex, skin color, age, live with mother, live with father, mother's schooling, residents of the house, cigarette smoking, drugs, physically aggression by an adult at home, involved in a fight with a firearm, involved in a fight with a melee weapon, suffered physical aggression, got involved in a fight, body satisfaction, health perception, computer at home, internet at home, full school, boarding school, have a cell phone, type of school

## Discussion

The present study investigated the association between clusters of obesogenic behaviors and social isolation in a population-based sample of Brazilian adolescents. We observed that the likelihood of loneliness and having close friends varied across the clusters, and adolescents from both healthier groups seem to have lower odds of loneliness.

The literature has shown that some psychosocial outcomes of adolescents can be influenced by obesogenic behaviors such as PA, diet, and SB [2, 11, 15]. In addition, there is evidence that adolescents with healthier obesogenic behavior profiles have better socialization, with lower chances of loneliness [5, 9, 10]. Our findings also highlight the fact that many adolescents have lifestyles defined by the coexistence of favorable and unfavorable health behaviors [16]. Thus, adolescents in clusters that include an active lifestyle seem to have more prosocial attitudes in their life, such as talking to friends and going for a walk more often [17], improving friendship quality and decreasing the chances of being socially isolated [4].

A systematic review investigating the relationship between friendship and PA analyzing cross-sectional, longitudinal, and experimental studies with adolescents in the United States showed that peers and friends play an important role in adolescents' PA levels. This association was positive in terms of peer support, presence of peers and/or friends, peer norms, friendship quality, peer acceptance and affiliation, and negative for peer victimization [18]. Despite the inverse chain to our proposed analyses, having friends helps to provide intrapersonal and interpersonal psychological support, favoring better psychological disposition for the maintenance of PA behavior during adolescence. In addition, being active favor socialization, evidencing a cyclical relationship between behaviors—socialization—behavior.

Some mediators have been hypothesized to help explain our findings. For instance, increasing self-confidence and supporting greater problem-solving ability were observed in the univariate relationships between PA and social isolation [17] and between SB and social isolation [5]. We hypothesize that PA and SB may share psychosocial mediators, and one behavior “supplies” the other in maintaining more prosocial attitudes. In other words, a cluster that lacks PA but the adolescent has a reduced time in sedentary behavior can still provide some benefits and vice-versa. Furthermore, obese adolescents report more loneliness, have fewer friends, and are more bullied [19]. In this case, it is suggested that healthier clusters can favor both better results for biological outcomes, such as decreasing overweight and obesity and minimizing interpersonal and intrapersonal vulnerabilities in adolescence [2].

Some limitations can be observed in this study. First, the cross-sectional nature of the data does not allow for a causal relationship to be established between adolescents' behaviors and our selected outcomes. The causal chain of these relationships between behavior and health outcomes is well established. However, it is plausible that the observed associations are bidirectional. Although obesity seems to influence social isolation, we ask for caution when interpreting our findings, as there was no control for this variable (not available for PeNSE—sample 1) in our adjusted analysis. Feeling lonely and having fewer friends can keep adolescents away from healthier behaviors, such as PA during leisure time. The social isolation construct is self-reported based on two indicators that may not represent the phenomenon's complexity; therefore, caution in interpreting the results is necessary. Nevertheless, the observed research problem is substantial. It has an analytical approach centered on the individual (clusters analysis); the clusters observed in this population were validated in previous research and involve a representative sample. To the authors’ knowledge this is the first study to assess the relationship between clusters of obesogenic behaviors and social isolation. In addition, this study draws attention to a less obvious association between lifestyle and health outcomes. Schools, administrators, and policymakers can look beyond obesity and realize that opportunities, attitudes, and choices (in non-particular order) about adolescent lifestyle can impact the social life and the mental health of young people. Therefore, multicomponent interventions are required, considering the synergies between lifestyle behaviors.

## Conclusions

Adolescents in clusters where health-favorable behaviors outweighed unfavorable ones were less likely to perceive themselves as lonely and without close friends. Clusters of obesogenic behaviors seem to share mediators that expose or protect adolescents not only to obesity but also to critical psychosocial outcomes for social life, such as having more friends.

## Supplementary Information

**Additional file 1: Table 1.** Continuation of the adolescent characteristics table, with behavioral variables, victimization and health outcomes. 

## Data Availability

The original PeNSE data set is publicly available in: https://www.ibge.gov.br/estatisticas/sociais/educacao/9134-pesquisa-nacional-de-saude-do-escolar.html?=&t=downloads

## Abbreviations

PA: Physical Activity
SB: Sedentary Behavior
PeNSE: National Adolescent School-based Health Survey
BIC: Schwarz’s Bayesian Criterion
OR: Odds Ratios
CI: Confidence Intervals
CONEP: Comissão Nacional de Ética em Pesquisa – CONEP

## References

[1] Biddle SJH, Ciaccioni S, Thomas G, Vergeer I (2019). Physical activity and mental health in children and adolescents: an updated review of reviews and an analysis of causality. Psychol Sport Exerc.

[2] Matias TS, Lopes MVV, de Mello GT, Silva KS (2019). Clustering of obesogenic behaviors and association with body image among Brazilian adolescents in the national school-based health survey (PeNSE 2015). Preventive Medicine Reports.

[3] Boone-Heinonen J, Gordon-Larsen P, Adair LS (2008). Obesogenic Clusters: Multidimensional Adolescent Obesity-related Behaviors in the U.S. Ann Behav Med.

[4] Yi X, Liu Z, Qiao W, Xie X, Yi N, Dong X (2020). Clustering effects of health risk behavior on mental health and physical activity in Chinese adolescents. Health Qual Life Outcomes.

[5] Werneck AO, Collings PJ, Barboza LL, Stubbs B, Silva DR (2019). Associations of sedentary behaviors and physical activity with social isolation in 100,839 school students: the Brazilian scholar health survey. Gen Hosp Psychiatry.

[6] Hoare E, Skouteris H, Fuller-Tyszkiewicz M, Millar L, Allender S (2014). Associations between obesogenic risk factors and depression among adolescents: a systematic review: obesogenic risk and depression: a review. Obes Rev.

[7] Leech RM, McNaughton SA, Timperio A (2014). The clustering of diet, physical activity and sedentary behavior in children and adolescents: a review. Int J Behav Nutr Phys Act.

[8] Matias TS, Silva KS, da Silva JA, de Mello GT, Salmon J (2018). Clustering of diet, physical activity and sedentary behavior among Brazilian adolescents in the national school - based health survey (PeNSE 2015). BMC Public Health.

[9] Hoare E, Milton K, Foster C, Allender S (2016). The associations between sedentary behaviour and mental health among adolescents: a systematic review. Int J Behav Nutr Phys Act.

[10] Padial-Ruz R, González-Campos G, Zurita-Ortega F, Puga-González ME (2020). Associations between feelings of loneliness and attitudes towards physical education in contemporary adolescents according to sex, and physical activity engagement. IJERPH.

[11] Iannotti RJ, Wang J (2013). Patterns of Physical Activity, Sedentary Behavior, and Diet in U.S. Adolescents. J Adolesc Health.

[12] Matias TS, Bacil EDA, Viero V dos SF, Vieira YP, da Silva LS, Sá AM, do Amaral CS, Cavazzotto TG. Clustering of Obesogenic Behaviors Associated With Bullying Roles Among 100,794 Adolescents. J Interpers Violence. 2022;0(0). 10.1177/08862605221132785. 10.1177/08862605221132785 36398924

[13] Gasparini L, Cruces G, Tornarolli L (2011). Recent trends in income inequality in Latin America. Economía.

[14] Brazilian Institute of Geography and Statistics. National School-Based Health Survey 2015. Rio de Janeiro: IBGE; 2016.

[15] Liberali R, Del Castanhel F, Kupek E, de Assis MAA (2021). Latent class analysis of lifestyle risk factors and association with overweight and/or obesity in children and adolescents: systematic review. Child Obes.

[16] de Mello GT, Lopes MVV, Minatto G, da Costa RM, Matias TS, Guerra PH (2021). Clustering of physical activity, diet and sedentary behavior among youth from low-, middle-, and high-income countries: a scoping review. IJERPH.

[17] Ferrar K, Chang C, Li M, Olds TS (2013). Adolescent time use clusters: a systematic review. J Adolesc Health.

[18] Fitzpatrick C, Burkhalter R, Asbridge M (2019). Adolescent media use and its association to wellbeing in a Canadian national sample. Preventive Medicine Reports.

[19] Waasdorp TE, Mehari K, Bradshaw CP (2018). Obese and overweight youth: risk for experiencing bullying victimization and internalizing symptoms. Am J Orthopsychiatry.

