# Supplementary material for: Clustering of physical activity, sedentary behavior, and diet associated with social isolation among brazilian adolescents
Source: BMC Public Health. 2023 Mar 25;23:562. doi: 10.1186/s12889-023-15444-x (PMC10039485; doi:10.1186/s12889-023-15444-x)
Supplement: Supplementary file 1 — Additional file 1: Table 1. Continuation of the adolescent characteristics table, with behavioral variables, victimization and health outcomes. [file 12889_2023_15444_MOESM1_ESM.docx]

**Supplementary material**

**Table 1.** Continuation of the adolescent characteristics table, with behavioral variables, victimization and health outcomes.

| Variables | n | % | 95% IC* |
| --- | --- | --- | --- |
| ***Behavioral factors*** |  |  |  |
| Cigarette smoking in the last 30 days |  |  |  |
| Do not smoke | 83,349 | 81.69 | 81.09 - 82.27 |
| Not once | 13,247 | 12.72 | 12.3 - 13.16 |
| 1-2 | 2,884 | 2.98 | 2.75 - 3.23 |
| 3-5 | 858 | 0.94 | 0.83 - 1.08 |
| 6-9 | 465 | 0.47 | 0.38 - 0.58 |
| 10-19 | 374 | 0.37 | 0.31 - 0.44 |
| 20-29 | 230 | 0.21 | 0.16 - 0.27 |
| Everyday | 606 | 0.59 | 0.51 - 0.68 |
| Used drugs in the last 30 days |  |  |  |
| do not use | 93,416 | 91.08 | 90.62 - 91.52 |
| did not use | 4,699 | 5.00 | 4.47 - 5.07 |
| 1-2 | 1,992 | 2.00 | 1.89 - 2.26 |
| 3-5 | 729 | 0.77 | 0.67 - 0.88 |
| 6-9 | 365 | 0.39 | 0.32 - 0.48 |
| >10 | 829 | 0.9 | 0.79 - 1.03 |
| Alcohol consumption in the last 30 days |  |  |  |
| do not drink | 47,008 | 44.56 | 43.75 - 45.38 |
| did not drink | 32,356 | 31.67 | 31.04 - 32.3 |
| 1-2 | 13,773 | 14.31 | 13.91 - 14.71 |
| 3-5 | 3,905 | 4.05 | 3.82 - 4.29 |
| 6-9 | 2,176 | 2.33 | 2.17 - 2.51 |
| 10-19 | 1,487 | 1.75 | 1.591 - 1.931 |
| 20-29 | 722 | 0.76 | 0.66 - 0.87 |
| Everyday | 534 | 0.55 | 0.47 - 0.65 |
| Involved in a fight with a firearm in the last 30 day |  |  |  |
| Yes | 5,583 | 5.73 | 5.41 - 6.08 |
| No | 95,735 | 94.26 | 93.91 - 94.59 |
| Involved in a fight with a melee weapon in the last 30 days |  |  |  |
| Yes | 8,118 | 7.91 | 7.57 - 8.27 |
| No | 93,066 | 92.08 | 91.73 - 92.43 |
| Got involved in a fight in the last 12 months |  |  |  |
| Not once | 78,332 | 76,64 | 76.04 - 77.23 |
| 1 | 11,419 | 11.92 | 11.53 - 12.32 |
| 2-3 | 5,719 | 5.76 | 5.49 - 6.04 |
| 4-5 | 2,099 | 2.08 | 1.92 - 2.25 |
| 6-7 | 1,050 | 1.05 | 0.94 - 1.16 |
| 8-9 | 595 | 0.64 | 0.55 - 0.73 |
| 10-11 | 450 | 0.43 | 0.36 - 0.51 |
| >12 | 1,418 | 1.47 | 1.33 - 1.63 |
| ***Victimization and health outcomes*** |  |  |  |
| Physically aggression by an adult at home in the last 30 days |  |  |  |
| Not once | 86,795 | 85.5 | 85.02 - 85.96 |
| 1 | 6,571 | 6.69 | 6.34 - 7.015 |
| 2-3 | 3,043 | 2.99 | 2.79 - 3.19 |
| 4-5 | 1,310 | 1.34 | 1.22 - 1.48 |
| 6-7 | 862 | 0.85 | 0.75 - 0.97 |
| 8-9 | 668 | 0.72 | 0.64 - 0.81 |
| 10-11 | 481 | 0.51 | 0.43 - 0.6 |
| >12 | 1,380 | 1.01 | 1.27 - 1.54 |
| Suffered physical aggression in the last 12 months |  |  |  |
| Not once | 82,986 | 81.64 | 81.12 - 82.16 |
| 1 | 7,963 | 8 | 7.65 - 8.36 |
| 2-3 | 4,712 | 4.67 | 4.41 - 4.94 |
| 4-5 | 1,931 | 1.95 | 1.78 - 2.13 |
| 6-7 | 1,000 | 0.96 | 0.86 - 1.07 |
| 8-9 | 577 | 0.56 | 0.49 - 0.65 |
| 10-11 | 454 | 0.47 | 0.4 - 0.56 |
| >12 | 1,533 | 1.71 | 1.57 - 1.85 |
| Body satisfaction |  |  |  |
| very satisfied | 28,480 | 28.89 | 28.19 - 29.59 |
| satisfied | 43,800 | 43.16 | 42.49 - 43.82 |
| indifferent | 10,614 | 10.28 | 9.93 - 10.65 |
| dissatisfied | 13,644 | 13.35 | 12.91 - 13.8 |
| very dissatisfied | 4,329 | 4.32 | 4.09 - 4.56 |
| Health perception |  |  |  |
| very good | 37,830 | 38.6 | 37.95 - 39.26 |
| Good | 34,625 | 34.41 | 33.8 - 35.03 |
| Regular | 20,980 | 19.87 | 19.39 - 20.36 |
| Bad | 4,342 | 38.72 | 3.671 - 4.084 |
| very bad | 3,541 | 32.46 | 3.034 - 3.471 |
